# Platelets correlate with false negative T-SPOT.TB results by inhibiting interferon-γ production in T cells *via* degranulation

**DOI:** 10.3389/fcimb.2022.937416

**Published:** 2022-08-24

**Authors:** Jiayue Rao, Yuting Rao, Yang Guo, Mei Jiang, Dan Long, Qing Luo, Zikun Huang, Junming Li

**Affiliations:** Department of Clinical Laboratory, The First Affiliated Hospital of Nanchang University, Nanchang, Jiangxi, China

**Keywords:** T cells, T-SPOT.TB, platelets, tuberculosis, interferon-gamma

## Abstract

**Background:**

T-SPOT.TB (T-SPOT) is widely used for the detection of Mycobacterium tuberculosis infection by detecting interferon-gamma (IFN-γ) release in T lymphocytes. This assay is performed on peripheral blood mononuclear cells (PBMCs) separated by Ficoll density gradient centrifugation, which often contain some residual platelets. Here, we investigated the impact of platelets on T-SPOT assay and related mechanisms.

**Methods:**

The correlation between platelet count, platelet-to-lymphocyte ratio (PLR), and the IFN-γ secreting T cells (ISCs) in positive control wells of T-SPOT assay were retrospectively analyzed. T-SPOT assay was performed with un-treated PBMCs, platelets-removed PBMCs, and platelets-enriched PBMCs to confirm the impact of platelets on T-SPOT assay. The activation of platelets and their impact on IFN-γ production in T cells were detected by flow cytometry (FCM). Platelets and T cells were cultured in a mixed culture system and co-culture system respectively, followed by detection of the frequencies of IFN-γ-producing T cells and the levels of intracellular IFN-γ in T cells by FCM. Moreover, the effect of platelet releasate on the T-SPOT assay was evaluated.

**Results:**

The ISCs in positive control wells of the T-SPOT assay showed a significant decrease with the increase in platelet count. The PLR of the peripheral blood were negatively correlated with the ISCs in positive control wells of the T-SPOT assay. Removal or enrichment of platelets significantly increased or decreased the ISCs and the positive rate of T-SPOT. Inhibition of platelet activation significantly increased the ISCs of T-SPOT. The frequencies of IFN-γ-producing T cells in PBMCs and the levels of intracellular IFN-γ were significantly reduced by the addition of platelets, both in the mixed culture system and the co-culture system. Platelet releasate upon thrombin activation significantly decreased the ISCs of T-SPOT.

**Conclusions:**

Platelets correlate with negative T-SPOT results by inhibiting IFN-γ production in T cells *via* degranulation.

## Introduction

Tuberculosis (TB) remains a leading infectious cause of death globally. World Health Organization (WHO) estimates that there were approximately 10 million new cases of TB worldwide in 2020 (World Health Organization [WHO], 2021), of which reactivation of latent TB infection (LTBI) is a major driver (Sharma et al., 2012). Approximately 2 billion people have LTBI and approximately 5-10% of them will develop active TB in their lifetime (World Health Organization [WHO], 2020). Nevertheless, the probability of developing active TB is greatly increased among people who are immunocompromised, such as those with autoimmune diseases, cancers, diabetics, or HIV infection. Therefore, it is essential to identify individuals with LTBI in these at-risk populations (Balbi et al., 2018; Ho and Leung, 2018; Lee et al., 2018).

Currently, interferon-gamma (IFN-γ) release assays (IGRAs) are the main approaches used for the identification of LTBI, which depend on measuring IFN-γ released by antigen-specific T cells in response to stimulation by *Mycobacterium tuberculosis* (Mtb) antigens. IGRAs can be divided into QuantiFERON-TB Gold In-Tube (QFT-GIT) assay and T-SPOT.TB (T-SPOT) assay, depending on the methods of specimen treatment and IFN-γ detection. T-SPOT assay is performed on peripheral blood mononuclear cells (PBMCs) separated by Ficoll density gradient centrifugation. Afterward, PBMCs are incubated with ESAT-6 and CFP-10 peptides to stimulate the release of IFN-γ and the result of T-SPOT is reported as the number of IFN-γ secreting T cells (ISCs). Therefore, to obtain reliable detection results, it is crucial to obtain high-quality PBMCs in the T-SPOT assay.

However, the PBMCs separated by Ficoll density gradient centrifugation often contain amounts of residual platelets, especially in individuals with thrombocytosis. In addition, TB patients and some other patients with a high risk of TB activation are sometimes accompanied by high platelet counts, such as patients with rheumatoid arthritis, myeloproliferative neoplasms, and cancer (Hutchinson et al., 1976; Renshaw and Gould, 2013; Spivak, 2017; Haemmerle et al., 2018). Moreover, accumulating evidence indicates that platelets can affect T cell function *in vitro* (Mudd et al., 2016; Dewi et al., 2020; Tan et al., 2022). Platelet count in whole blood have been studied as a risk factor for QFT-GIT and T-SPOT assay (Kim et al., 2011; Li et al., 2019), however, no study has been reported on the effect of platelets mixed in PBMCs on T-SPOT assay.

In this study, we investigated whether these platelets mixed in PBMCs affect the T-SPOT assay. Our results show that platelets can significantly downregulate the positive rate of the T-SPOT assay by inhibiting IFN-γ production in T cells through degranulation. This study lays a foundation for improving the accuracy of the currently used T-SPOT assay.

## Materials and methods

### Subjects and samples

TB Patients were recruited from the Jiangxi Chest Hospital and the First Affiliated Hospital of Nanchang University and were confirmed by smear microscopy, Mtb culture, or GeneXpert TB assay. Healthy donor controls were healthy men and women, 25–70 years old. All subjects with pregnancy, autoimmune diseases, cancer, HIV infection, diabetes mellitus, chronic renal failure, chronic liver disease were excluded. This study was approved by the Ethical Review Committees of the First Affiliated Hospital of Nanchang University (ethical review No.003) and was carried out in compliance with the Helsinki Declaration. Written informed consent was obtained from all the subjects enrolled in this study. All experiments are carried out in the biosafety level 2 laboratory.

### Cell isolation

PBMCs were isolated from the peripheral blood samples using Ficoll density gradient centrifugation as described previously (Su et al., 2019).

For platelet isolation, the peripheral blood samples were centrifuged for 6 min at 180 g and the upper two-thirds of platelet-rich plasma (PRP) was collected. PRP was further centrifuged at 330 g for 15 min to obtain platelet pellets.

The removal of platelets from PBMCs was performed as follows: the peripheral blood sample was centrifuged for 6 min at 180 g to obtain PRP. PRP was further centrifuged at 2200g for 10min, the upper layer of plasma is collected, and the platelet sediment was discarded. Then, the platelet-poor plasma is added back to the peripheral blood and the PBMCs were isolated from the platelet-poor blood.

The enrichment of platelets in PBMCs was performed as follows: the peripheral blood sample was centrifuged for 6 min at 180 g to obtain PRP. The upper PRP was sucked out with a sterile pipette. Half of the remaining cell fraction without PRP was discarded and a half was retained. The PRP was further centrifuged at 330 g for 15 min and half of the upper layer of plasma was discarded. Then, the remaining PRP was mixed with the remaining cell fraction to reconstitute the platelet-rich whole blood, followed by the isolation of PBMCs from the platelet-enriched blood.

### Flow cytometry analysis

Cells were incubated with FcR blocking reagent for 5 min followed by incubating with or without the mixture of fluorescent-labeled anti-human monoclonal antibodies for 30min: platelets were identified with anti-CD41-PE MAb (Beckman Coulter, USA); platelet activation was monitored by anti-CD62P-FITC MAb (eBioscience, USA); T cells were identified by anti-CD3-PC7 MAb (Beckman Coulter, USA). Perfix-nc Kit (Beckman Coulter, USA) and anti-IFN-γ-PE (Beckman Coulter, USA) were used for intracellular staining. Annexin V-FITC and propidium iodide were used to eliminate the dead cells from the analysis. Auto-fluorescence and nonspecific staining were determined by using isotype-matched controls. The flow cytometry (FCM) analysis was performed using a Cytomics FC 500 flow cytometer (Beckman Coulter, USA).

### T-SPOT assays

The T-SPOT assay (Oxford Immunotec Ltd., Abingdon, UK) was performed according to the manufacturer’s instructions. Briefly, heparin-anticoagulated peripheral venous blood was collected and processed within 4 hours. PBMCs were isolated by Ficoll density gradient centrifugation, washed, and counted. ESAT-6, CFP-10, and phytohaemagglutinin (PHA) as a positive control, and an equal volume of AIM-V medium as a negative control, were added to the microplate wells, respectively. Subsequently, PBMCs were added into the wells at 2.5 x 10^5^ cells per well. After incubation for 18-20 hours, secondary antibodies and substrates were added to form visible spots. Spot counts were performed using a vSPOT ELISPOT reader (Autoimmun Diagnostika GmbH, Germany). Results are expressed as spot-forming cells or IFN-γ-secreting cells (ISCs). The threshold for a positive result was six spots in any well stimulated with ESAT-6 or CFP-10 and at least twice the number of spots as in the negative control in the T-SPOT assay.

### Statistical analysis

Values were presented as mean ± standard deviation (SD) and analyzed using GraphPad Prism 9.0 software. Shapiro-Wilk normality test was used to examine normal distribution. Independent Samples t-test or paired t-test were used when normal data distribution was confirmed, and Mann-Whitney U-test or Wilcoxon signed-rank test was used for the variables without normal distribution. The Chi-square test was sued for the comparison of frequencies between groups. Differences were considered statistically significant at P < 0.05.

## Results

### Platelet count and platelet-to-lymphocyte ratio in peripheral blood correlate with the amount of IFN-γ secreting T cells (ISCs) in T-SPOT assay

**Figure 1:**
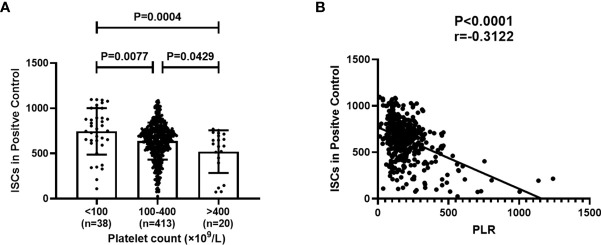
Platelet Count and platelet-to-lymphocyte ratio (PLR) correlates with the amount of ISCs in positive control wells of the T-SPOT assay. 471 subjects were divided into three groups according to platelet count and the spot counts in positive control in these three groups were compared. PLR in peripheral blood was counted and the correlation between PLR and the spot counts in positive control was evaluated. **(A)** Each symbol denotes a single subject, and the mean ± SD for each study population is shown. Statistical significance was determined by one-way analysis of variance (ANOVA)followed by Tukey’s multiple comparisons test. **(B)** Correlation analysis was performed using Spearman’s rho correlations and each symbol denotes a single subject.

In the positive control well of the T-SPOT assay, cells are incubated with a non-specific stimulator, PHA. The spot counts in positive control reflect the PHA IFN-γ response and are independent of the prevalence of the subjects. To investigate the correlation between the platelet count in peripheral blood and the ISCs in positive control well, 471consecutive subjects who received T-SPOT detection were divided into three groups according to the platelet count, platelet count less than 100(n=38), platelet count between 100 and 400 (n=413), platelet count above 400(n=20). Then, ISCs reflected by the spot counts in positive control wells of these three groups were compared. In addition, the PLR was counted and the correlation between PLR and the ISCs in positive control wells was evaluated. Results showed that the ISCs in positive control wells showed a significant decrease with the increase in platelet count (**Figure 1A**, P=0.0004), and the PLR was negatively correlated with the spot counts in positive control. (**Figure 1B**, P=<0.001, r=-0.3122).

### Platelet count in PBMCs fraction was positively correlated with platelet count in peripheral blood

**Figure 2:**
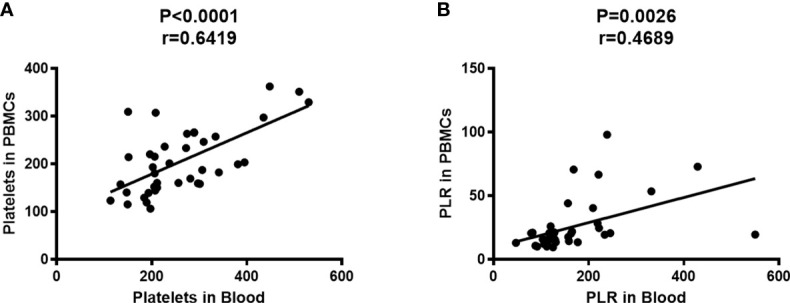
Platelet count in PBMCs was positively correlated with that in peripheral blood. The platelet counts and the lymphocyte counts in peripheral blood and PBMCs were measured by an automatic blood cell analyzer (n=39). Correlation analysis was performed using Spearman’s rho correlations. **(A)** The number of platelets in PBMCs was positively correlated with that in peripheral blood (P<0.0001, r=0.6419). **(B)** The PLR in PBMCs was positively correlated with that in peripheral blood (P=0.0026, r=0.4689).

Subsequently, the correlation between platelet counts in whole blood and PBMCs fraction was investigated. PBMCs were isolated from peripheral blood by Ficoll density gradient centrifugation, followed by measurement of platelet count and lymphocyte count in peripheral blood and PBMCs using an automatic blood cell analyzer (System, Japan). The results show that the platelet count in PBMCs was positively correlated with that in peripheral blood (**Figure 2A**, P<0.0001, r=0.6419), meanwhile, the PLR in PBMCs was positively correlated with that in peripheral blood (**Figure 2B**, P=0.0026, r=0.4689).

### Removal of platelets from PBMCs fraction increased the ISCs and the positive results of the T-SPOT assay

**Figure 3:**
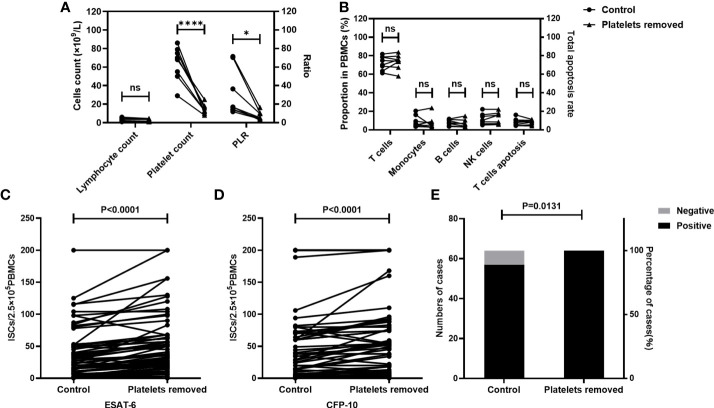
The numbers of ISCs and the positive results of T-SPOT were increased after the removal of platelets. The platelet count, the apoptosis rate of T cells, and the proportion of cells in non-platelet-removed PBMC sand platelet-removed PBMCs were measured (n=8). T-SPOT assays were performed with the non-platelet-removed PBMCs and platelets-removed PBMCs with the same numbers of mononuclear cells(n=64). **(A, B)** The lymphocyte count, the platelet count, the PLR, the apoptosis rate of T cells, and the proportion of cells in PBMCs in two groups were compared by paired Student’s t-test. *p<0.1, ****P<0.0001, ns, not significant. The ISCs in response to **(C)** ESAT-6 and **(D)** CFP-10 stimulation were counted and compared by paired Student’s t-test. **(E)** The positive rates of the T-SPOT assay were compared by the chi-square test.

Firstly, the effect of platelet removal on the proportion of cells in PBMCs, and T cell viability were investigated. Platelet-removed PBMCs and non-platelet-removed PBMCs were isolated (n=8). The lymphocyte count and platelet count were detected by an automatic blood cell analyzer, and the apoptosis of T cells and the proportion of cell subsets in PBMCs were measured by FCM. Results showed that the platelet count and the PLR significantly decreased after platelet removal, while the lymphocyte count, the rate of T cell apoptosis, and the proportion of each cell subsets in PBMCs did not change significantly before and after platelet removal (**Figures 3A, B**).

Subsequently, the impact of platelet removal on the T-SPOT assay was investigated. Peripheral blood was collected from TB patients (n=64) and the whole blood from each individual was divided into two parts. In one part of peripheral blood, PBMCs were isolated directly. In another part of peripheral blood, PBMCs were isolated after removal of platelets. Then, T-SPOT assay was performed with these PBMCs and the number of PBMCs was adjusted to 2.5×10^5^/well. Results showed that after the removal of platelets, the numbers of the ISCs significantly increased in response to both ESAT-6 (**Figure 3C**, P<0.0001) and CFP-10 (**Figure 3D**, P<0.0001) stimulation. In addition, the positive rate of the T-SPOT assay increased from 89.06% to 100.00% after the removal of platelets (**Figure 3E**, P=0.0131).

### Enrichment of platelets decreased the ISCs of T-SPOT assay and the positive results of the T-SPOT assay

**Figure 4:**
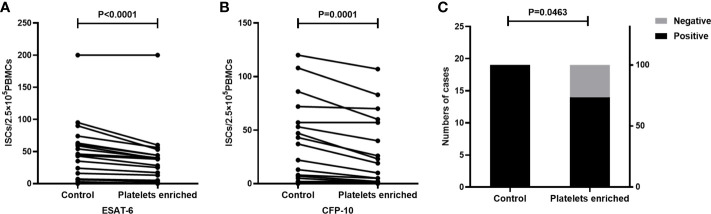
The enrichment of platelets decreased the ISCs in the T-SPOT assay. T-SPOT assays were performed with non-platelet-enriched PBMCs and platelets-enriched PBMCs with the same numbers of mononuclear cells (n=19). The ISCs in response to **(A)** ESAT-6 and **(B)** CFP-10 stimulation were counted and compared by paired Student’s t-test. **(C)** The positive rates of the T-SPOT assay were compared by the chi-square test.

Peripheral blood was collected from TB patients(n=19) and the whole blood from each individual was divided into two parts, one of which was directly detected by the T-SPOT assay. Another part of the peripheral blood was used to prepare platelet enriched specimen as described in the method section, followed by PBMC isolation and T-SPOT detection. The number of PBMCs used for the T-SPOT assay in these two parts was adjusted to 2.5×10^5^/well. Results showed that when compared to the untreated group, platelet enrichment significantly decreased the numbers of ISCs both in response to ESAT-6 (**Figure 4A**, P<0.001) and CFP-10 (**Figure 4B**, P=0.0001). The positive rate of T-SPOT assay detected with platelet enriched samples was also significantly decreased (**Figure 4C**, P=0.0463).

### The process of PBMCs extraction activates platelets

**Figure 5:**
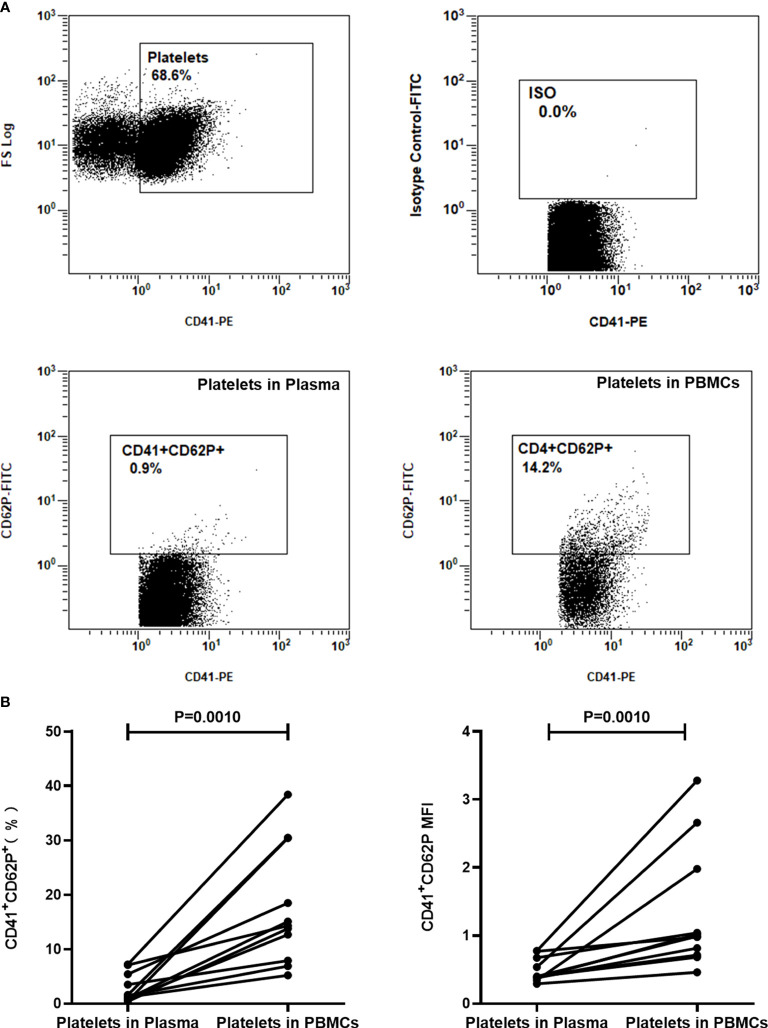
The expression of CD62P on platelets in PBMCs was significantly increased than that in plasma layers. The expression of CD62P in platelets from uncentrifuged plasma layers and platelets from PBMCs separated by density gradient centrifugation was detected by FCM (n=9). **(A)** Representative flow cytometry scatter plots of the expression of CD62P on platelets. Platelets were gated as CD41^+^ population. **(B)** The expression of CD62P on platelets in plasma and PBMCs were compared by paired Student’s t-test.

We next focused on understanding the molecular mechanisms of platelets inhibiting the production of IFN-γ. Activation is the basis for platelets to exert their immune function. To determine whether the platelets in PBMCs would be activated by the isolation procedure, the expression of CD62P on platelets in plasma layers and platelets in PBMC fractions prepared by density gradient centrifugation was detected by FCM (n=9). Data showed that the expression of activation marker CD62P on platelets in PBMCs was significantly increased than that in plasma (**Figure 5**, P=0.0010).

### Inhibition of platelets activation increased ISCs and the positive results of the T-SPOT assay

**Figure 6:**
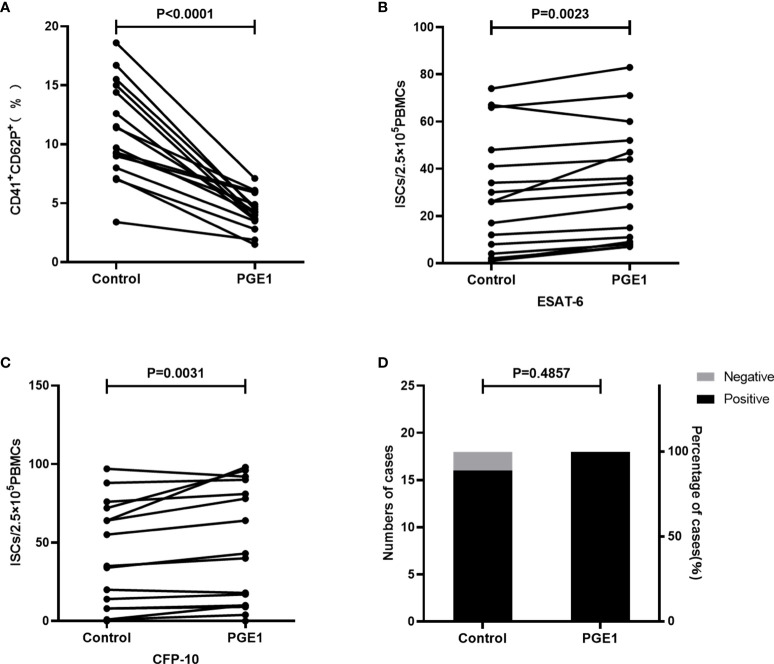
The numbers of ISCs and the positive results of T-SPOT were increased after inhibition of platelet activation. PBMCs were isolated and PGE1 was used to prevent platelet activation during the isolation procedure, followed by PBMCs being used for the T-SPOT assay (n=16). **(A)** The expression of CD62P on platelets in PBMCs before and after PGE1 treatment was measured by FCM and compared by paired Student’s t-test. The ISCs in response to **(B)** ESAT-6 and **(C)** CFP-10 stimulation were counted and compared by paired Student’s t-test. **(D)** The positive rates of the T-SPOT assay were compared by the chi-square test.

To further validate the effect of platelet activation on T-SPOT assay, the Ficoll solution used for density gradient centrifugation was supplemented with 100nM prostaglandin E1(PGE1) to prevent platelet activation during the isolation procedure of PBMCs. Briefly, peripheral blood was collected from TB patients (n=16) and the whole blood from each individual was divided into two parts and used for PBMCs isolation respectively, one of which by using the Ficoll solution mixed with PGE1 at the final concentration of 100nM and the other using the untreated Ficoll solution. These two resulting PBMCs were then used for T-SPOT detection with the same numbers of mononuclear cells. Results showed that the expression of CD62P on platelets in PBMCs was significantly decreased after treatment with PGE1, indicating that platelet activation was inhibited (**Figure 6A**, P<0.0001). When compared to the untreated group, inhibition of platelet activation significantly increased the numbers of ISCs both in response to ESAT-6 (**Figure 6B**, P=0.0023) and CFP-10 (**Figure 6C**, P= 0.0031). Moreover, although no statistical difference was found because of the few negative specimens, the positive rate of the T-SPOT assay increased from 88.88% to 100% after inhibition of platelet activation (**Figure 6D**).

### Platelets suppress IFN-γ production in T cells

**Figure 7:**
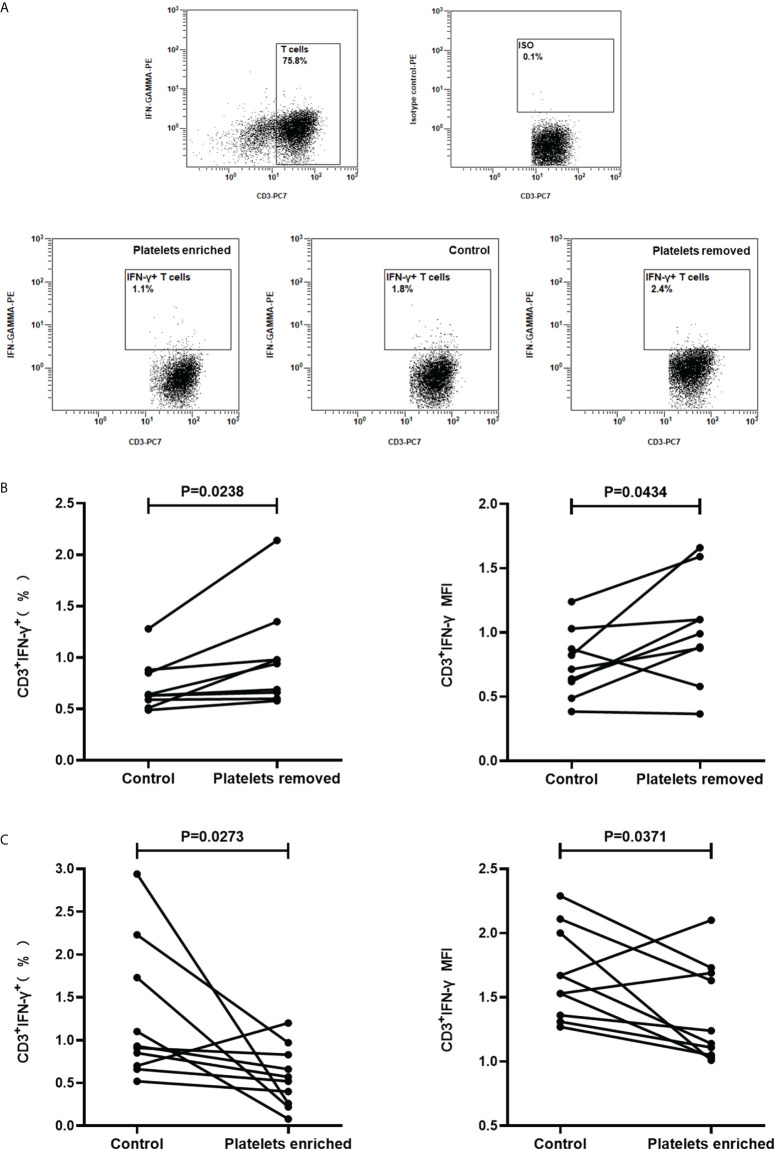
Regulating the frequencies of platelets in PBMCs significantly affects the production of IFN-γ in T cells. Platelets-removed PBMCs (n=9) or platelets-enriched PBMCs (n=9) were stimulated with PHA and the production of IFN-γ in T cells was measured by FCM. **(A)** Representative flow cytometry scatter plots of IFN-γ production in T cells. **(B, C)** The frequencies of IFN-γ-producing T cells and the mean fluorescence intensity (MFI) upon platelet removal or platelet enrichment were compared with the control group by paired Student’s t-test.

Considering that the main target cells of the T-SPOT assay are T cells, the above results suggested that activated platelets can suppress the IFN-γ production in T cells. To further clarify this hypothesis, peripheral blood was collected from healthy volunteers, followed by the isolation of platelets-removed PBMCs (n=9) or platelets-enriched PBMCs (n=9). Then, PBMCs were stimulated with PHA and the intracellular IFN-γ in T cells was measured by FCM (**Figure 7A**). Results showed that the removal of platelets increased the frequency of IFN-γ-producing T cells (**Figure 7B**, P=0.0238), as well as the levels of intracellular IFN-γ in T cells which are reflected by the mean fluorescence intensity (MFI) (**Figure 7B**, P=0.0434). In addition, the enrichment of platelets significantly decreased the frequency of IFN-γ-producing T cells (**Figure 7C**, P=0.0273) as well as the levels of intracellular IFN-γ in T cells (**Figure 7C**, P=0.0371).

### Platelets suppress IFN-γ production in T cells in a non-contact dependent manner

**Figure 8:**
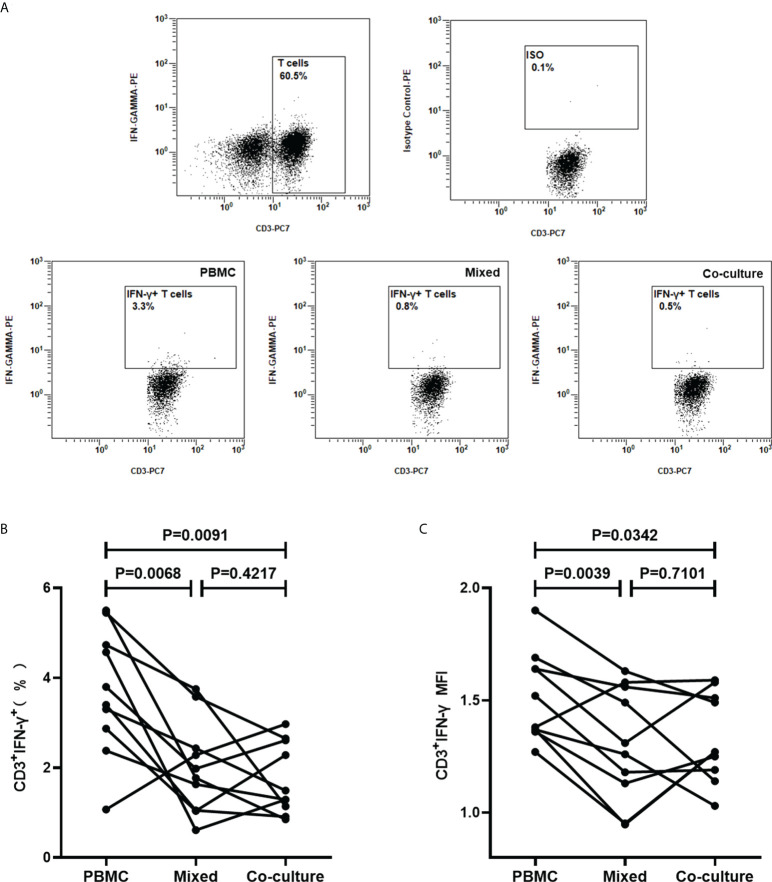
Platelets suppress IFN-γ production in T cells in a non-contact-dependent manner. PBMCs were cultured and stimulated with PHA for 16 h, in the absence of platelets (PBMC), or the presence of platelets in mixed culture system (Mixed) or co-culture system (Co-culture), followed by the detection of the intracellular IFN-γ in T cells by FCM (n=10). **(A)** Representative flow cytometry scatter plots of IFN-γ production in T cells. **(B, C)** The frequencies of IFN-γ-producing T cells and the MFI of three groups were compared by paired Student’s t-test.

It was next questioned whether the effects of platelets on the T cells are contact-dependent. Platelets and PBMCs, in the ratio of 100, were mixed cultured or co-cultured in a transwell system with a 0.4 µm transwell insert, followed by stimulation with PHA for 16h and detected for the intracellular IFN-γ in T cells by FCM (n=10). It was demonstrated that the frequencies of IFN-γ-producing T cells and the levels of intracellular IFN-γ were significantly reduced by the addition of platelets, both in the mixed culture system (P=0.0068, P=0.0039) and the co-culture system (P=0.0091, P=0.0342) (**Figure 8**). The frequencies of IFN-γ-producing T cells (P=0.4217) and the levels of intracellular IFN-γ(P=0.7101) in the co-culture system did not show a significant difference when compared to the mixed culture system (**Figure 8**).

### Platelet releasate decreased the ISCs of T-SPOT assay

**Figure 9:**
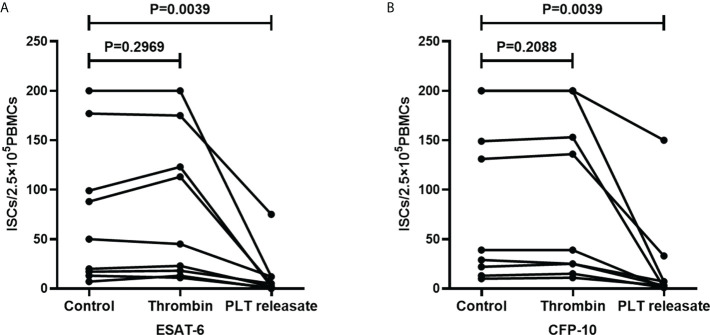
The supernatants of thrombin-activated platelets decreased the numbers of ISCs. PBMCs used for T-SOPT assay were resuspended with AIM-V medium, the supernatants of thrombin-activated platelets, or AIM-V medium mixed with thrombin, respectively (n=9). The ISCs of three groups in response to **(A)** ESAT-6 and **(B)** CFP-10 were counted and compared by paired Student’s t-test.

Platelets release many immunoregulatory soluble mediators in response to activation stimuli (Manne et al., 2017). To investigate the impact of platelet releasate on T-SPOT assay, purified platelets in the AIM-V medium were activated with thrombin for 1h and the supernatants were collected. PBMCs were isolated from freshly collected peripheral blood of TB patients and divided into three parts, one part was resuspended with AIM-V medium, and one part was resuspended with the supernatants of thrombin-activated platelets, and the other part was resuspended with AIM-V medium with thrombin. Then, PBMCs were used for T-SOPT detection with the same numbers of mononuclear cells (n=9). Results showed that when compared to the untreated group, thrombin did not influence the numbers of ISCs. However, the supernatants of thrombin-activated platelets significantly decreased the numbers of ISCs both in response to ESAT-6 (**Figure 9A**, P=0.0039) and CFP-10 (**Figure 9B**, P= 0.0039).

## Discussion

T-SPOT assay is a commonly used method to detect Mtb infection. In this assay, ESAT-6 and CFP-10 peptides are used to stimulate and activate the antigen-specific T cells in PBMCs, followed by detecting the amounts of ISCs by the ELISPOT platform. Previous studies have reported that false-negative result is one of the main problems of T-SPOT assay, in which some risk factors for the false-negative result have been reported, such as increased age, over-weight, HIV co-infection, serous effusion, and so on (Pan et al., 2015; Zhang et al., 2017; Shangguan et al., 2020).

Based on the cell-mediated immunity in PBMCs, the T-SPOT assay is susceptible to the activity, purity, and quantity of PBMCs. However, the PBMCs separated by density gradient centrifugation always mix with residual platelets. In the present study, the PLR in peripheral blood was found negatively correlated with the spot counts in Positive Control. Moreover, the count of the platelets mixed in PBMCs was positively correlated with the platelet count in peripheral blood. These findings hinted to us that these platelets mixed in PBMCs may affect the accuracy of the T-SPOT assay. To verify this hypothesis, T-SPOT assays were performed with platelets-removed PBMCs and platelets-enriched PBCMs, while ensuring that the number of assayed mononuclear cells was the same in each group. Herein, the results confirmed that the removal of platelets had little effect on the lymphocytes count, the ratio of each type of cells, and the apoptosis rate of T cells in PBMCs. In addition, the number of ISCs and the positive rate of T-SPOT assay increased significantly after the removal of platelets from PBMCs and decreased significantly after the enrichment of platelets in PBMCs. These results revealed that the platelet mixed in PBMCs can reduce the sensitivity of the T-SPOT assay.

Platelets are known to process immunomodulatory properties and a prerequisite for them to play the immunomodulatory role is to be activated. In our study, it was observed that the expression of activation marker CD62P on platelets in PBMCs was significantly increased than on platelets in uncentrifuged plasma layers, indicating that some platelets were activated during the process of isolating PBMCs. In addition, we found that inhibition of platelet activation in PBMCs can increase ISCs and the positive results of the T-SPOT assay, demonstrating that platelets can affect the T-SPOT assay after being activated.

T cell is the main source of ISCs (Wang et al., 2016). In the current study, we found that, in response to PHA stimulation, the enriched platelets significantly decreased the frequency of IFN-γ-producing T cells, while the removal of platelets significantly increased the frequency of IFN-γ-producing T cells. Together, these results confirmed that the platelet mixed in PBMCs can inhibit IFN-γ production in T cells and thus affecting the T-SPOT assay. This is in agreement with the recent studies, which showed that the platelets cocultured with T cells exert an inhibitory impact on the production of IFN-γ in T cells (Zamora et al., 2017; Polasky et al., 2020; Zimmer et al., 2020).

Upon activation, platelets can secrete numerous soluble inflammatory mediators and/or bind to leukocytes, thus exerting an immune effect (Rachidi et al., 2017; Zamora et al., 2017; Polasky et al., 2020; Zimmer et al., 2020). In the present study, transwell assays confirmed that the regulation of IFN-γ production in T cells by platelets is mainly mediated by soluble mediators, as the production of IFN-γ of T cells in cultures with the spatial separation of PBMC and platelets by transwell insert significantly decreased compared to the cultures in the absence of platelets but did not show a significant difference compared to the cultures with direct platelets to PBMCs contact. Furthermore, it was observed that the supernatants of thrombin-activated platelets significantly decreased the numbers of ISCs in the T-SPOT assay, confirming that platelets can exert inhibitory effects on the T-SPOT assay by soluble factors. However, it remains to be further investigated which soluble mediators play a role in the effect of platelets on the T-SPOT assay.

To our knowledge, no previous study has investigated the role of platelets mixed in PBMCs in T-SPOT assay. The current study, for the first time, uncovers that the platelets mixed in PBMCs can suppress the IFN-γ production of T cells and decrease the sensitivity of the T-SPOT assay. Furthermore, we demonstrated that platelets exert their immunosuppressive function on T cells after activation mainly *via* platelet-released soluble mediators. This study suggests that negative T-SPOT results may not be reliable in patients with high platelet counts. For patients with highly suspected Mtb infection, T-SPOT should be retested after removing platelets.

## Data availability statement

The raw data supporting the conclusions of this article will be made available by the authors, without undue reservation.

## Ethics statement

The studies involving human participants were reviewed and approved by Ethical Review Committees of the First Affiliated Hospital of Nanchang University. The patients/participants provided their written informed consent to participate in this study.

## Author contributions

JL and ZH conceived and design the experiment. JR performed the experiments. YR, YG, and MJ contributed significantly to the acquisition of data, analysis, and interpretation of data. JR drafted the manuscript. DL and QL helped perform the analysis with constructive discussions. All the authors have accepted responsibility for the entire content of this submitted manuscript and approved submission.

## Fundings

This work was supported by the project for high and talent of Science and Technology Innovation in Jiangxi “double thousand plan” (Grant No. jxsq2019201024), grants from the National Natural Science Foundation of China (Grant No. 82160308), the Project for Academic and Technical Leaders of Major Disciplines of Jiangxi Province (Grant No. 20172BCB22026), the Natural Science Foundation of Jiangxi Province of China (Grant No. 20212BAB216028).

## Conflict of interest

The authors declare that the research was conducted in the absence of any commercial or financial relationships that could be construed as a potential conflict of interest.

## Publisher’s note

All claims expressed in this article are solely those of the authors and do not necessarily represent those of their affiliated organizations, or those of the publisher, the editors and the reviewers. Any product that may be evaluated in this article, or claim that may be made by its manufacturer, is not guaranteed or endorsed by the publisher.
